# 

**Published:** 1904-06

**Authors:** 


*THE BRISTOL MEDICO-CHIRURGICAL SOCIETY.*

CONTENTS.

| | PAGE |
|---|---|
| **Forms of Joint Disease met with in Medical Practice.** By WM. HALE WHITE, M.D. Lond., F.R.C.P. ... ... | 97 |
| **Notes on some Cases from an Electrical Department.** By GEORGE PARKER, M.A., M.D. Camb. ... ... ... ... ... | 112 |
| **A Case of Cæsarean Section for Obstruction of Labour by a Pelvic Tumour.** By W. C. SWAYNE, M.D., B.S. Lond. ... | 120 |
| **A Case of Congenital Hypertrophic Stenosis of the Pylorus.** By THEODORE FISHER, M.D. Lond., M.R.C.P., and NEWMAN NEILD, M.B., Ch.B. Vict. ... | 123 |
| **A Case of Æstivo-Autumnal Malarial Fever with Parasites of an Unusual Type.** By J. O. SYMES, M.D. Lond., D.P.H. | 126 |
| **Notes on a Case of Blackwater Fever.** By E. CECIL WILLIAMS, B.A., M.B. Cantab. ... ... ... ... ... ... ... | 128 |
| **Progress of the Medical Sciences:** | |
| **Medicine.** G. PARKER, M.D. Cantab. ... | 131 |
| **Surgery.** C. A. MORTON ... ... ... ... | 136 |
| **Obstetrics.** W.C. SWAYNE, M.D., B.S. Lond. | 140 |
| **Reviews of Books** ... ... ... ... ... ... | 145 |
| **Editorial Notes** ... ... ... ... ... ... | 175 |
| **Notes on Preparations for the Sick** ... | 181 |
| **The Library of the Bristol Medico-Chirurgical Society** ... ... ... ... | 185 |
| **Meetings of Societies** ... ... ... ... ... | 188 |
| **Local Medical Notes** ... ... ... ... ... | 191 |

